# Supplementary material for: Access and use of oxytocin for postpartum haemorrhage prevention: a pre-post study targeting the poorest in six Mesoamerican countries
Source: BMJ Open. 2020 Mar 16;10(3):e034084. doi: 10.1136/bmjopen-2019-034084 (PMC7076242; doi:10.1136/bmjopen-2019-034084)
Supplement: Author's manuscript [file bmjopen-2019-034084.draft_revisions.pdf]

BMJ Open

BMJ Open is committed to open peer review. As part of this commitment we make the peer review history of every article we publish publicly available.

When an article is published we post the peer reviewers' comments and the authors' responses online. We also post the versions of the paper that were used during peer review. These are the versions that the peer review comments apply to.

The versions of the paper that follow are the versions that were submitted during the peer review process. They are not the versions of record or the final published versions. They should not be cited or distributed as the published version of this manuscript.

BMJ Open is an open access journal and the full, final, typeset and author-corrected version of record of the manuscript is available on our site with no access controls, subscription charges or pay-per-view fees (http://bmjopen.bmj.com).

If you have any questions on BMJ Open's open peer review process please email info.bmjopen@bmj.com

BMJ Open

# Access and use of oxytocin for post-partum hemorrhage prevention: targeting the poorest in six Mesoamerican countries

| | |
|---|---|
| Journal: | *BMJ Open* |
| Manuscript ID | bmjopen-2019-034084 |
| Article Type: | Original research |
| Date Submitted by the Author: | 04-Sep-2019 |
| Complete List of Authors: | Kamath, Aruna; Institute for Health Metrics and Evaluation; University of Washington Department of Anesthesiology and Pain Medicine,<br>Schaefer, Alexandra; Institute for Health Metrics and Evaluation, Global Health<br>Palmisano, Erin; Institute for Health Metrics and Evaluation, Global Health<br>Johanns, Casey; Institute for Health Metrics and Evaluation, Global Health<br>Gonzalez Marmol, Alvaro; Inter-American Development Bank<br>Dinarte Mendoza, Mauricio ; Inter-American Development Bank<br>Schwarzbauer, Karla ; Inter-American Development Bank<br>Zúñiga-Brenes , Paola; Inter-American Development Bank<br>Ríos-Zertuche , Diego; Inter-American Development Bank<br>Iriarte, Emma; Inter-American Development Bank<br>mokdad, ali; Institute for Health Metrics and Evaluation<br>Hernandez Prado, Bernardo; Institute for Health Metrics and Evaluation |
| Keywords: | Maternal medicine < OBSTETRICS, International health services < HEALTH SERVICES ADMINISTRATION & MANAGEMENT, PUBLIC HEALTH |
| | |

SCHOLARONE™
Manuscripts

# Access and use of oxytocin for post-partum hemorrhage prevention: targeting the poorest in six Mesoamerican countries

Aruna M. Kamath,[1,2] Alexandra M. Schaefer,[1] Erin B. Palmisano,[1] Casey K. Johanns,[1] Alvaro Gonzalez Marmol,[3] Mauricio Dinarte Mendoza,[3] Karla Schwarzbauer,[3] Paola Zúñiga-Brenes,[3] Diego Rios-Zertuche,[3] Emma Iriarte,[3] Ali H. Mokdad,[1] Bernardo Hernández[1]

1. Institute for Health Metrics and Evaluation, University of Washington, Seattle, WA, USA
2. Department of Anesthesiology, University of Washington, Seattle, WA, USA
3. Salud Mesoamérica Initiative/Inter-American Development Bank, Panama, Panama; Tegucigalpa, Honduras; San José, Costa Rica; and Washington, DC, USA

**Corresponding Author:**

Aruna M. Kamath, MD, MPH
Institute for Health Metrics and Evaluation
University of Washington
2301 Fifth Ave, Suite 600
Seattle, WA 98121
USA
Tel: 206.897.2817
Email: akamath@uw.edu

For peer review only

## ABSTRACT

**Introduction:** Hemorrhage remains the leading cause of maternal mortality both worldwide and in Mesoamerica. Active management of third stage of labor and oxytocin use to reduce postpartum hemorrhage mortality are not optimally applied in Mesoamerica. The Salud Mesoamérica Initiative aims to reduce maternal mortality in Mesoamerica.

**Methods**: We compared the availability and administration of oxytocin for the prevention of postpartum hemorrhage in the poorest Mesoamerican communities. 166 basic-level and comprehensive-level health facilities in Belize, Guatemala, Honduras, Mexico, Nicaragua, and Panama were included in the study. Medical records were sampled at random using a systematic sampling method for uncomplicated full-term deliveries (n = 2,470) at baseline (2011–2013) and at first-phase follow-up (2013–2014). Multivariable logistic regression analysis was used.

**Results**: Availability of oxytocin increased from 82.9% to 97.6%. Oxytocin administration increased from 83.6% to 88.4%. Significant improvements were seen for availability of oxytocin (aOR = 8.41, 95% CI = 1.50–47.30). Administration of oxytocin was found to be significantly higher in Honduras (aOR = 2.96; 95% CI = 1.00-8.76) in reference to Guatemala at follow-up.

**Conclusion**: After interventions to increase health facility supplies, the study showed a significant improvement in availability but not administration of oxytocin in poor communities within Mesoamerica. Efforts are needed to improve the use of oxytocin.

## STRENGTHS AND LIMITATIONS OF THIS STUDY

- To our knowledge, this study is the first multinational study of oxytocin coverage amongst this targeted poor population and region. We provide a current assessment of the evidence-to-practice gap for post-partum hemorrhage prevention in Mesoamerica for the most underserved populations.



For peer review only - http://bmjopen.bmj.com/site/about/guidelines.xhtml

- Challenges to this study involve logistic limitations in collecting data on some of the facility inputs.

# INTRODUCTION

Hemorrhage remains the leading cause of maternal mortality both worldwide and in Mesoamerica, accounting for 31.4% of total maternal deaths globally(1) and ranging from 38.0% of total maternal deaths in Nicaragua to 17.9% in Belize based on recent estimates.(2) While maternal mortality has declined in the last two decades, Millennium Development Goal 5, of reducing maternal mortality by three-fourths, was not achieved globally in 2015.(3,4) A new target has been set for the Sustainable Development Goals to reduce the global maternal mortality ratio to less than 70 per 100,000 live births by 2030.(5) Currently, most of the Mesoamerican countries in this study fall short of or near this target, with the highest maternal mortality ratio, of 109.6 per 100,000 live births, occurring in Honduras.(6)

International guidelines for postpartum hemorrhage, from the World Health Organization (WHO), International Federation of Gynecology and Obstetrics (FIGO), and the International Confederation of Midwives (ICM), recommend active management of third stage of labor (AMTSL) with emphasis on administering oxytocin, the drug of choice for prevention and treatment of uterine atony.(7,8) Oxytocin is the cornerstone of AMTSL due to its efficacy in reducing postpartum hemorrhage risk by 40–60%.(9–11) Despite such evidence, translating these best practices into a real-world clinical standard of care is not optimally applied in Mesoamerica, with the proper use of uterotonics as low as 10–20% in some studies.(12,13)

With efforts to close the evidence-to-practice gap, the Salud Mesoamérica Initiative (SMI) aims to reduce maternal and child mortality for the poorest quintile in Mesoamerica.(14) SMI is a public-private partnership between the Ministries of Health in participating Mesoamerican countries and donors that focuses on improving  preventive child health, family planning, antenatal care,



For peer review only - http://bmjopen.bmj.com/site/about/guidelines.xhtml

peripartum care, and Essential Obstetric and Newborn Care (EONC).(15) SMI follows a results-based financing model (16,17) in which Ministries of Health commit to achieve negotiated targets for eight to twelve performance indicators. To receive the performance payment incentive, countries need to achieve at least eighty percent of targets. At the beginning of each phase, donors contribute approximately half the funding, with the rest from domestic sources.

The first phase of SMI interventions, presented in this study, aims to improve system readiness by increasing availability of inputs and improving norms. The second phase targets improvements in coverage and quality of care. Specifically, performance indicators for postpartum hemorrhage prevention involve increasing availability of oxytocin in this first phase and ensuring administration of oxytocin postpartum in the second phase.

In this study, a multinational analysis of oxytocin use for this targeted population and region, we examine the availability and administration of oxytocin for the prevention of postpartum hemorrhage at the first follow-up of the SMI intervention. In doing so, we assess the extent to which international guidelines are adhered to on the patient level for one aspect of essential obstetric care in Mesoamerica.

# METHODS

## Study setting and design

As part of the Salud Mesoamérica Initiative, 2,470 uncomplicated deliveries from 166 health facilities were included in this analysis to assess the availability and administration of oxytocin for the prevention of postpartum hemorrhage in the poorest Mesoamerican communities. These poorest communities were identified by the Initiative administrators based on census data, using as criteria to have the highest concentration of population in the lowest quintile of income.  Data



For peer review only - http://bmjopen.bmj.com/site/about/guidelines.xhtml

collection was conducted at the baseline (2011–2013) and the first-phase follow-up (2013–2014) time periods.

The first phase, a year of intervention implementation prior to follow-up data collection, focused on system readiness and supply-side performance indicators. For postpartum hemorrhage prevention, the performance indicator involved increasing the availability of oxytocin, through interventions such as strengthening supply chains; improving procurement processes, warehouse and pharmacy storage practices; traffic light systems to monitor expiry dates; inventory management processes; improving norms and protocols of care to adhere to international guidelines; creating decision flow-charts and checklists.

Facilities were classified into different levels of Essential Obstetric and Neonatal Care (EONC):(15) basic-level facilities have the capacity to manage uncomplicated vaginal deliveries and stabilize patients with complications prior to transfer to higher level of care; and comprehensive-level facilities attend both uncomplicated and complicated births, with capacity to accept referral patients and perform surgical and emergency care. While the project and its evaluation includes all countries in Mesoamerica, due to availability of data for this specific analysis we used data from Ministry of Health facilities serving the poorest areas in Belize, Guatemala, Honduras, Mexico (state of Chiapas), Nicaragua, and Panama. Basic- and comprehensive-level health facilities were selected with certainty due to a small number of these hospitals. Because of this, the same facilities were visited in the baseline and follow-up measurements.

A three-part health facility survey was administered at these facilities for both rounds of data collection. The survey included an interview questionnaire to the facility directors on the facility infrastructure and resources; an observation checklist of pharmaceutical inventory and medical and laboratory equipment; and a retrospective review of medical records to examine treatment practices. Medical records of uncomplicated deliveries per international classification of diseases (ICD) coding during the specified timeframes were sampled at random using a systematic sampling



For peer review only - http://bmjopen.bmj.com/site/about/guidelines.xhtml

method, until the required quota for each facility level was met. For the systematic sampling, using the listing of deliveries attended in each facility, a random starting point was selected in time over the period of study (about 18 months), and from there cases were selected with an interval equal to the size of the sampling fraction. Sampling quotas varied by facility type, round, and country. Sample size of records to be reviewed was calculated due to availability of resources. Expected sample sizes had enough power to detect differences in evaluation indicators, including treatment to deliveries according to the national norms. Due to availability of resources, it was possible to increase the number of cases in the sample in the follow-up to increase power, especially in the countries with a reduced number of cases in the baseline (Belize). Overall, we had a power over 80% to detect differences of 10 percentage points between baseline and follow-up for oxytocin availability in all countries excepting Belize and Nicaragua. Further details of the methodology are available elsewhere.(14,17–19)

Quality of data in medical records varied by facility and country. To measure standardized, replicable, and comparable metrics from varying medical record sources, indicators that were linked to discharge diagnosis and ICD coding were constructed by the Ministries of Health based on the protocols and guidelines of each country. Criteria checklists for each indicator differed by EONC facility level. These criteria checklists were then transformed into data points and conditional algorithms for data collection. During field visits, medical record data availability and measurability of criteria were assessed.(20) Data collection was conducted by trained physicians and nurses from the region, and data were submitted electronically using the survey software DatStat Illume.

Availability of oxytocin was defined as supply on the day of survey visit; and administration of oxytocin was defined as given intravenously or intramuscularly for postpartum hemorrhage prevention (excluding postpartum hemorrhage treatment).(20) Uncomplicated delivery records selected for review included full-term (>37 weeks) gestational age at participating health facilities, including cesarean sections without complications per ICD coding. Deliveries with adverse outcomes



For peer review only - http://bmjopen.bmj.com/site/about/guidelines.xhtml

per ICD coding that required hemorrhage treatment were excluded, due to collection under a separate survey module.

Approval for this study involved the institutional review board from the University of Washington (exemption as a non-human-subject research determination), partnering data-collection agencies, and the Ministry of Health in each country. Prior to data collection, informed consent was obtained from each health facility administrator. During the data extraction process, medical records were anonymized.

## Statistical analysis

We used multivariable logistic regression analysis to evaluate possible facility-level factors associated with the primary outcomes of oxytocin availability and administration. Covariates included in the analysis were timing of data collection (baseline versus first-phase follow-up), country, and EONC facility type (basic versus comprehensive). Additionally for oxytocin administration, we examined relevant training within the last year (routine labor care, basic emergency obstetrical care, or maternal complications care); and oxytocin availability (day of survey visit), and skilled personnel (doctor, nurse). When fitting the regression models, skilled personnel was dropped from the model due to predicting success perfectly, meaning a skilled personnel was always present for the administration of oxytocin. The rate of missing data was 4.4% for oxytocin availability and 0.7% for oxytocin administration for the regression analysis. P values <0.05 were considered significant. Stata 14.2 (StataCorp LP, College Station, TX, USA) was used for all analysis.



For peer review only - http://bmjopen.bmj.com/site/about/guidelines.xhtml

# RESULTS

## Sample characteristics

Table 1 presents the medical records, patient, facility, and personnel characteristics. The majority of deliveries were in basic-level health facilities (64.3%). We sampled the most medical records from Guatemala (27.5%), followed by Chiapas (Mexico) (20.8%), Honduras (19.6%), Panama (16.0%), Nicaragua (11.9%), and Belize (4.2%), based on the quota for records within each facility, prevalence of records of interest, and sampling interval. Mean maternal age was 24.3 years and ranged from 11 to 48 years. Of these women, 55.2% were single, 44.8% were married or in a social partnership, and 72.7% had less than a secondary education. Nearly all patients (99.7%) had a single gestational pregnancy, and only 0.3% had a multiple gestational pregnancy. Most of these patients delivered vaginally (97.7%) as opposed to by cesarean section (2.3%). Supply (on the day of survey visit) for oxytocin at baseline was 82.9%, which increased to near complete availability (97.6%) at first-phase follow-up. Personnel on staff – that is, skilled birth attendants employed by the facility – at baseline was 96.2% for physicians and 91.0% for nurses and increased to 100% and 93.2%, respectively, at follow-up. Relevant training – that is, instruction on routine labor care, basic emergency obstetrical care, or maternal complications care – provided within the last year increased from 74.0% at baseline to 92.0% at first-phase follow-up.

For peer review only

## Table 1. Medical records, patient, and facility characteristics by first-phase follow-up

| **Medical records characteristics** | | | | |
|---|---|---|---|---|
| | | **n (%)** | | |
| | | **Baseline**<br>922 (37.3) | **Follow-up**<br>1,548 (62.7) | **Total**<br>2,470[a] (100) |
| | EONC[b] facility type | | | |
| | Basic-level | 616 (66.8) | 971 (62.7) | 1,587 (64.3) |
| | Comprehensive-level | 306 (33.2) | 577 (37.3) | 883 (35.8) |
| | Country | | | |



For peer review only - http://bmjopen.bmj.com/site/about/guidelines.xhtml

| | | | |
|---|---|---|---|
| Belize | 14 (1.5) | 90 (5.8) | 104 (4.2) |
| Guatemala | 247 (26.8) | 432 (27.9) | 679 (27.5) |
| Honduras | 234 (25.4) | 249 (16.1) | 483 (19.6) |
| Mexico | 180 (19.5) | 334 (21.6) | 514 (20.8) |
| Nicaragua | 90 (9.8) | 205 (13.2) | 295 (11.9) |
| Panama | 157 (17.0) | 238 (15.4) | 395 (16.0) |
| **Patient characteristics** | | | |
| Maternal age[c] | 24.3 (6.7) | 24.3 (6.6) | 24.3 (6.6) |
| Marital status | | | |
| Single | 409 (61.8) | 656 (51.8) | 1,065 (55.2) |
| Partnership | 253 (38.2) | 610 (48.1) | 863 (44.8) |
| Education | | | |
| Less than secondary | 392 (70.5) | 772 (73.9) | 1,164 (72.7) |
| Secondary or higher | 164 (29.5) | 273 (26.1) | 437 (27.3) |
| Pregnancy type | | | |
| Single gestational | 154 (98.7) | 917 (99.9) | 1,071 (99.7) |
| Multiple gestational | 2 (1.3) | 1 (0.1) | 3 (0.3) |
| Delivery type | | | |
| Vaginal | 409 (98.6) | 1,091 (97.3) | 1,500 (97.7) |
| Cesarean section | 6 (1.5) | 30 (2.7) | 36 (2.3) |
| **Facility and personnel characteristics** | | | |
| | **n (%)** | | |
| | **Baseline** 78 (47.0) | **Follow-up** 88 (53.0) | **Total** 166 (100) |
| Oxytocin supply[d] | 58 (82.9) | 83 (97.6) | 141 (91.0) |
| Personnel on staff[e] | | | |
| Physician | 75 (96.2) | 88 (100) | 163 (98.2) |
| Nurse | 71 (91.0) | 82 (93.2) | 153 (92.2) |
| Relevant training[f] | 57 (74.0) | 81 (92.0) | 138 (83.6) |

a n may vary for each variable due to missingness

b Essential Obstetric and Newborn Care

c mean ± SD

d day of survey visit

e skilled birth attendants employed by the facility. Midwife excluded due to varying skill level amongst countries.

f within last year, includes routine labor care, basic emergency obstetrical care, maternal complications

## Availability and administration of oxytocin for postpartum hemorrhage prevention

Overall, availability of oxytocin (on the day of the survey) increased from 82.9% to 97.7% and administration of oxytocin increased from 83.6% to 88.4% from baseline to first-phase follow-up. Notably, all countries achieved 100% availability of oxytocin at first-phase follow-up, except for Guatemala (91.7). Comprehensive level facilities achieved 100% availability, compared to basic level

For peer review only



For peer review only - http://bmjopen.bmj.com/site/about/guidelines.xhtml

facilities (96.8%) at first-phase follow-up. Administration of oxytocin at first-phase follow-up ranged from 80.0% in Belize to 94.8% in Honduras. Oxytocin administration was nearly the same at basic (88.4%) and comprehensive level (88.5%) facilities at first-phase follow-up. (Table 2).

## Table 2. Oxytocin availability and administration by facility type, country, and first-phase follow-up

| Oxytocin Availability | | n (%) | | |
|---|---|---|---|---|
| | | **Baseline** 58 (82.9) | **Follow-up** 83 (97.6) | **Total**[a] 141 (91.0) |
| EONC facility type[b] | | | | |
| | Basic-level | 43 (84.3) | 61 (96.8) | 104 (91.2) |
| | Comprehensive-level | 15 (78.9) | 22 (100.0) | 37 (90.2) |
| Country | | | | |
| | Belize | 4 (100.0) | 4 (100.0) | 8 (100.0) |
| | Guatemala | 15 (88.2) | 22 (91.7) | 37 (90.2) |
| | Honduras | 13 (92.9) | 12 (100.0) | 25 (96.2) |
| | Mexico | 7 (100.0) | 14 (100.0) | 21 (75.0) |
| | Nicaragua | 6 (85.7) | 14 (100.0) | 20 (95.2) |
| | Panama | 13 (92.9) | 17 (100.0) | 30 (96.8) |
| **Oxytocin Administration for prevention of post-partum hemorrhage** | | **n (%)** | | |
| | | **Baseline** 771 (83.6) | **Follow-up** 1,353 (88.4) | **Total** 2,124 (86.6) |
| EONC facility type | | | | |
| | Basic-level | 521 (84.6) | 852 (88.4) | 1,373 (86.9) |
| | Comprehensive-level | 250 (81.7) | 501 (88.5) | 751 (86.1) |
| Country | | | | |
| | Belize | 9 (64.3) | 72 (80.0) | 81 (77.9) |
| | Guatemala | 198 (80.2) | 393 (91.0) | 591 (87.0) |
| | Honduras | 224 (95.7) | 221 (94.8) | 445 (95.3) |
| | Mexico | 129 (71.7) | 277 (82.9) | 406 (79.0) |
| | Nicaragua | 87 (96.7) | 168 (82.8) | 255 (87.0) |
| | Panama | 124 (79.0) | 222 (93.3) | 346 (87.6) |

a n may vary for each variable due to missingness

b Essential Obstetric and Newborn Care

Table 3 shows possible first-phase follow-up and facility determinants of the main outcomes: the availability and administration of oxytocin. Odds of availability were significantly higher at first-phase follow-up compared to baseline (aOR = 8.41, 95% CI = 1.50–47.30), while odds of administration increased in magnitude but were not significant at first-phase follow-up (aOR = 1.63, 95% CI 0.83–3.21). Additionally, administration of oxytocin was found to be significantly higher in Honduras (aOR = 2.96; 95% CI = 1.00–8.76) in reference to Guatemala. Other covariates examined,

For peer review only



For peer review only - http://bmjopen.bmj.com/site/about/guidelines.xhtml

such as comprehensive-level facility, relevant training, and oxytocin availability, showed a positive but not significant correlation to the primary outcomes.

## Table 3. Factors associated with availability and administration of oxytocin

| | Oxytocin availability | | Oxytocin administration for prevention of post-partum hemorrhage | |
|---|---|---|---|---|
| | **Crude OR** (95% CI) (n= 2,257) | **Adjusted OR[a]** (95% CI) (n= 2,257) | **Crude OR** (95% CI) (n =2,343) | **Adjusted OR** (95% CI) (n =2,343) |
| First-phase follow-up | **7.29 (1.48–35.84)** | **8.41 (1.50–47.30)** | 1.43 (0.79–2.62) | 1.63 (0.83–3.21) |
| Country | | | | |
| Belize | *Predicts success perfectly[b]* | *Predicts success perfectly* | 0.52 (0.13–2.12) | 0.44 (0.11–1.71) |
| Guatemala | 1 | 1 | 1 | 1 |
| Honduras | 1.70 (0.18–16.46) | 2.24 (0.27–18.24) | **2.71 (1.02–7.22)** | **2.96 (1.00–8.76)** |
| Mexico | 0.67 (0.14–3.10) | 0.51 (0.11–2.46) | 0.57 (0.25–1.31) | 0.57 (0.24–1.37) |
| Nicaragua | 2.22 (0.23–21.71) | 2.01 (0.19–20.92) | 1.00 (0.38–2.62) | 0.95 (0.35–2.54) |
| Panama | 3.43 (0.36–33.05) | 3.83 (0.36–41.22) | 1.04 (0.41–2.66) | 1.03 (0.40–2.61) |
| Comprehensive-level facility | 1.00 (0.24–4.15) | 1.29 (0.31–5.31) | 0.92 (0.47–1.81) | 0.96 (0.46–2.00) |
| Relevant training[c] | -- | -- | 1.49 (0.71–3.13) | 1.08 (0.44–2.67) |
| Oxytocin availability[d] | -- | -- | 2.01 (0.92–4.39) | 1.45 (0.66–3.19) |

a adjusted for first-phase follow-up, country, and comprehensive-level facility. Relevant training and oxytocin availability not included (not applicable).

b Belize attained availability of oxytocin at all times (regression for this binary variable not possible)

c within last year, includes routine labor care, basic emergency obstetrical care, maternal complications

d day of survey visit

All models clustered at health facility level

# DISCUSSION

## Main findings

To our knowledge, this study is the first multinational analysis of oxytocin use among the poorest population within Mesoamerica. We provide an updated, regional evaluation of postpartum hemorrhage prevention practices that may guide clinical management and policy priorities for this



For peer review only - http://bmjopen.bmj.com/site/about/guidelines.xhtml

targeted population. Our study showed that the availability of oxytocin improved significantly after system readiness interventions. Administration of oxytocin increased but not in the same proportion as availability, which suggests that increasing the availability of inputs is necessary but not sufficient to improve quality of care.

## Interpretation

There remains a paucity of studies with regard to oxytocin availability and use in Mesoamerica (13,21–25) or in low-resource countries in general.(12,27–32) In Mesoamerica, previous studies have evaluated hospitals within a single country(13,21,22) rather than through a multinational analysis, compared global regions without sub-analysis by country in each region,(26) or indirectly evaluated oxytocin use with relevant training being the primary outcome.(23–25) On a broader level, other studies compare oxytocin to other uterotonics worldwide,(31–35) and international guidelines have been revised.(36) However, oxytocin remains the first-line drug for postpartum hemorrhage prevention in health facilities. Thus, this study is of value in providing a current assessment of the implementation of active management of third stage of labor practices in Mesoamerica for the most underserved populations.

Our study attests that clinical standards of care could be achieved in Mesoamerica with proper monitoring and management of oxytocin supplies. As expected from the implementation timeline, the first stage of the Salud Mesoamérica Initiative focused on improving system readiness, namely strengthening facility infrastructure, supply chains, availability and stock monitoring for drugs and equipment, and referral networks; while the second stage is primarily focused on increasing health care coverage and quality of care through interventions such as training, service delivery reorganization, and quality improvement.(14,37) Consequently, increasing the availability of oxytocin, a goal of the first phase of the initiative, was achieved. Increasing the availability of inputs is necessary but not sufficient to improve quality of care. Comprehensive quality improvement strategies would be needed that involve not only increasing availability of equipment and supplies,



For peer review only - http://bmjopen.bmj.com/site/about/guidelines.xhtml

but also improving skills and abilities of personnel, and establishing mechanisms to improve processes, identify causes, and lift barriers to ensure proper practices of care in the next phase.

## Limitations

This study has some limitations. First, oxytocin availability was defined based on the day of the visit to health facilities, which may not match the day of delivery in a medical record. Due to logistic limitations, it was not possible to assess stock-outs of oxytocin over a longer period of time. Therefore, we must consider oxytocin availability the day of the visit as a proxy for availability over a longer period of time. Quality of available oxytocin cannot be included when defining oxytocin availability, as data was not collected on expiration or storage of oxytocin. Second, this study focused on uncomplicated full-term deliveries and did not include patients resulting in hemorrhage due to two separate survey modules for data collection. In the module with adverse outcomes from hemorrhage, uterotonics were often administered more than once without distinction of whether for prevention or treatment. Third, sample sizes also vary by country, and in some cases like Belize, the reduced sample size limited our capacity to have country-specific precise estimates and to detect significant differences between baseline and follow-up measurements. Fourth, quality of record keeping may affect the information used in this study and may vary by country. Nonetheless, we used a standard methodology across countries, automated data collection, and used quality control measures that ensure comparability between countries.

# CONCLUSION

The study showed a significant improvement in availability of oxytocin at facilities that provide care to poor communities within Mesoamerica, but not optimal administration, as expected after emphasis on supply-side interventions. Continued monitoring and evaluation, beyond input availability, are essential to better understand how to improve processes and clinical practices. Our



For peer review only - http://bmjopen.bmj.com/site/about/guidelines.xhtml

study provides valuable information to close the evidence-to-practice gap and to reduce maternal mortality in Mesoamerica.

# LIST OF ABBREVIATIONS

Active management of third stage of labor (AMTSL)

Essential Obstetric and Newborn Care (EONC)

International Confederation of Midwives (ICM)

International Federation of Gynecology and Obstetrics (FIGO)

Salud Mesoamérica Initiative (SMI)

World Health Organization (WHO)

# DECLARATIONS

## Ethics approval

University of Washington Institutional Review Board. No patient consent required.

## Patient and Public Involvement

Patients or the public were not involved in the design, conduct, reporting, or dissemination of our research.

## Data Sharing

Data are owned by the Inter-American Development Bank. SMI baseline survey data, which comprise the minimal dataset, are publicly available on the SMI website. SMI follow-up survey data are currently available upon written request from the Inter-American Development Bank, whose contact information is listed below. Javier Lesaca, Communications Officer, Salud Mesoamerica



For peer review only - http://bmjopen.bmj.com/site/about/guidelines.xhtml

Initiative, Inter-American Development Bank, 1300 New York Ave. NW, Washington, DC 20577. contact@saludmesoamerica.org.

## Competing interests

The authors declare that they have no competing interests

## Funding

Salud Mesoamérica Initiative (SMI) is a public-private partnership funded by the Bill & Melinda Gates Foundation (Grant Number OPPGH5328), Agencia Española de Cooperación Internacional para el Desarrollo (10.13039/501100004892), and the Carlos Slim Foundation (10.13039/100004429), through the Inter-American Development Bank. AMK is funded by the National Institutes of Health T32 grant (T32GM086270). Funders had no role in study design, data collection, data analysis and interpretation, or preparation of the manuscript.

## Authors' contributions

AMK conceived the study and prepared data analysis, interpretation of data, initial draft, and final manuscript. AMS, EBP, and CKJ contributed to and reviewed data analysis. AGM, MDM, KS, PZB, DRZ, and EI contributed to the acquisition of the data. BH and AHM supervised the study and data compilation and contributed to interpretation of data. All authors critically reviewed the draft and approved the final manuscript.

## Acknowledgments

The authors recognize our colleagues at the Inter-American Development Bank, the Institute for Health Metrics and Evaluation, and our collaborating data collection institutions across Mesoamerica: University of Belize in Belize, Fundación FES in Guatemala and Honduras, El Colegio de la Frontera Sur in Mexico and Nicaragua, Centro de Investigación y Estudios en Salud/Universidad de Nicaragua in Nicaragua, and UNIMER-Centroamerica in Guatemala, Mexico, Honduras, Nicaragua,



For peer review only - http://bmjopen.bmj.com/site/about/guidelines.xhtml

Panama, and Belize. We also acknowledge Adrienne Chew from the Institute for Health Metrics and Evaluations for editing this manuscript.

# REFERENCES


1. GBD 2016 Causes of Death Collaborators. Global, regional, and national age-sex specific mortality for 264 causes of death, 1980–2016: a systematic analysis for the Global Burden of Disease Study 2016. The Lancet. 2017 Sep 16;390(10100):1151–210.

2. Institute for Health Metrics and Evaluation (IHME). GBDCompareDataVisualization. Seattle, WA: IHME, University of Washington, 2016. [Internet]. [cited 2018 Aug 22]. Available from: http://vizhub.healthdata.org/gbd-compare.

3. United Nations. Millennium Development Goals Report 2013. [Internet]. 2013 [cited 2018 Mar 11]. Available from: http://www.un.org/millenniumgoals/pdf/report-2013/mdg-report-2013-english.pdf

4. Lozano R, Wang H, Foreman KJ, Rajaratnam JK, Naghavi M, Marcus JR, et al. Progress towards Millennium Development Goals 4 and 5 on maternal and child mortality: an updated systematic analysis. The Lancet. 2011 Sep 24;378(9797):1139–65.

5. Every Women Every Child. The global strategy for women's, children's, and adolescents' health (2016-2030) [Internet]. 2015 [cited 2018 Mar 11]. Available from: http://globalstrategy.everywomaneverychild.org/pdf/EWEC_globalstrategyreport_200915_FINAL_WEB.pdf

6. Kassebaum NJ, Barber RM, Bhutta ZA, Dandona L, Gething PW, Hay SI, et al. Global, regional, and national levels of maternal mortality, 1990–2015: a systematic analysis for the Global Burden of Disease Study 2015. The Lancet. 2016 Oct 8;388(10053):1775–812.

7. World Health Organization. WHO recommendations for the prevention and treatment of postpartum haemorrhage. [Internet]. 2012 [cited 2018 Mar 11]. Available from: http://apps.who.int/iris/bitstream/10665/75411/1/9789241548502_eng.pdf

8. Lalonde A, FIGO Safe Motherhood and Newborn Health (SMNH) Committee. Prevention and treatment of postpartum hemorrhage in low-resource settings. Int J Gynecol Obstet. 2012 May 1;117(2):108–18.

9. Prata N, Bell S, Weidert K. Prevention of postpartum hemorrhage in low-resource settings: current perspectives. Int J Womens Health. 2013;5:737–52.

10. Prendiville WJ, Harding JE, Elbourne DR, Stirrat GM. The Bristol third stage trial: active versus physiological management of third stage of labour. BMJ. 1988 Nov 19;297(6659):1295–300.

11. Elbourne DR, Prendiville WJ, Carroli G, Wood J, McDonald S. Prophylactic use of oxytocin in the third stage of labour. Cochrane Database Syst Rev. 2001;(4):CD001808.

12. Althabe F, Mazzoni A, Cafferata ML, Gibbons L, Karolinski A, Armbruster D, et al. Using Uniject to increase the use of prophylactic oxytocin for management of the third stage of labor in Latin America. Int J Gynaecol Obstet Off Organ Int Fed Gynaecol Obstet. 2011 Aug;114(2):184–9.


For peer review only



For peer review only - http://bmjopen.bmj.com/site/about/guidelines.xhtml

13. García-Elorrio E, Aleman A, Cafferata ML, Colomar M, Tomasso G, Lacayo Y, et al. A multifaceted intervention to increase prophylactic oxytocin use during the third stage of labor and to reduce routine episiotomies in Nicaragua. Int J Gynaecol Obstet Off Organ Int Fed Gynaecol Obstet. 2014 Oct;127(1):31–4.

14. Mokdad AH, Colson KE, Zúñiga-Brenes P, Ríos-Zertuche D, Palmisano EB, Alfaro-Porras E, et al. Salud Mesoamérica 2015 Initiative: design, implementation, and baseline findings. Popul Health Metr. 2015 Feb 7;13:3.

15. USAID MCHIP Essential Obstetric and Newborn Care (EONC) Program Implementation Guidance [Internet]. [cited 2018 Mar 20]. Available from: https://www.k4health.org/sites/default/files/EONC%20Program%20Implementation%20Guide%20v1.1.pdf

16. Regalia F, Eichler R, Gigli S, Cleason M, Tapia Conyer R, Hernandez G, et al. Not Your Cookie-Cutter Results-Based Aid Initiative: Salud Mesoamerica Initiative's Experience Improving Health for the Poorest in Mesoamerica [Internet]. Inter-American Development Bank; 2017 Oct [cited 2018 Jun 6]. Available from: http://publications.iadb.org/handle/11319/8567

17. Álvarez MC, Tapia Conyer R, Regalia F, Eichler R, Ciria Matilla MC, Nelson J, et al. External Measurement as a Catalyst for Change in a Regional Results-Based Aid Initiative - The Salud Mesoamerica Experience [Internet]. Inter-American Development Bank; 2017 Oct [cited 2018 Dec 12]. Available from: https://publications.iadb.org/handle/11319/8565

18. Mokdad AH, Palmisano EB, Zúñiga-Brenes P, Ríos-Zertuche D, Johanns CK, Schaefer A, et al. Supply-side interventions to improve health: Findings from the Salud Mesoamérica Initiative. PloS One. 2018;13(4):e0195292.

19. Duber HC, Hartford EA, Schaefer AM, Johanns CK, Colombara DV, Iriarte E, et al. Appropriate and timely antibiotic administration for neonatal sepsis in Mesoamérica. BMJ Glob Health. 2018 May 1;3(3):e000650.

20. Rios-Zertuche D, Zúñiga-Brenes P, Palmisano E, Hernández B, Schaefer A, Johanns CK, et al. Methods to measure quality of care and quality indicators through health facility surveys in low- and middle-income countries. Int J Qual Health Care [Internet]. [cited 2018 Dec 12]; Available from: https://academic-oup-com.offcampus.lib.washington.edu/intqhc/advance-article/doi/10.1093/intqhc/mzy136/5039910

21. Low Lisa Kane, Bailey Joanne Motiño, Sacks Emma, Medina Lilian, Piñeda Hector Oqueli Lopez. Postpartum Hemorrhage Prevention: A Case Study in Northern Rural Honduras. J Midwifery Womens Health. 2010 Dec 24;53(1):e1–6.

22. Low Lisa Kane, Bailey Joanne Motino, Sacks Emma, Robles Chayla, Medina Lilian. Reduced postpartum hemorrhage after implementation of active management of the third stage of labor in rural Honduras. Int J Gynecol Obstet. 2012 Sep 12;119(3):217–20.

23. Dresang Lee T., González María Mercedes Ancheta, Beasley John, Bustillo Maura Carolina, Damos Jim, Deutchman Mark, et al. The impact of Advanced Life Support in Obstetrics (ALSO) training in low-resource countries. Int J Gynecol Obstet. 2015 Aug 4;131(2):209–15.

24. Walton Anna, Kestler Edgar, Dettinger Julia C., Zelek Sarah, Holme Francesca, Walker Dilys. Impact of a low-technology simulation-based obstetric and newborn care training scheme on




For peer review only - http://bmjopen.bmj.com/site/about/guidelines.xhtml

non-emergency delivery practices in Guatemala. Int J Gynecol Obstet. 2015 Dec 11;132(3):359–64.

25. Figueras Albert, Narváez Edgar, Valsecia Mabel, Vásquez Susana, Rojas Germán, Camilo Angiolina, et al. An Education and Motivation Intervention to Change Clinical Management of the Third Stage of Labor—The GIRMMAHP Initiative. Birth. 2008 Nov 18;35(4):283–90.

26. Sheldon W, Blum J, Vogel J, Souza J, Gülmezoglu A, Winikoff B, et al. Postpartum haemorrhage management, risks, and maternal outcomes: findings from the World Health Organization Multicountry Survey on Maternal and Newborn Health. BJOG Int J Obstet Gynaecol. 2014 Mar 1;121:5–13.

27. Olmedo Benjamin, Miranda Eva, Cordon Oscar, Pettker Christian M., Funai Edmund F. Improving maternal health and safety through adherence to postpartum hemorrhage protocol in Latin America. Int J Gynecol Obstet. 2014 Jan 24;125(2):162–5.

28. Braddick Louise, Tuckey Victoria, Abbas Zan, Lissauer David, Ismail Khaled, Manaseki-Holland Semira, et al. A mixed-methods study of barriers and facilitators to the implementation of postpartum hemorrhage guidelines in Uganda. Int J Gynecol Obstet. 2015 Sep 28;132(1):89–93.

29. Hoogenboom G, Thwin MM, Velink K, Baaijens M, Charrunwatthana P, Nosten F, et al. Quality of intrapartum care by skilled birth attendants in a refugee clinic on the Thai-Myanmar border: a survey using WHO Safe Motherhood Needs Assessment. BMC Pregnancy Childbirth. 2015 Feb 5;15:17.

30. Nelissen Ellen, Ersdal Hege, Østergaard Doris, Mduma Estomih, Broerse Jacqueline, Evjen-Olsen Bjørg, et al. Helping mothers survive bleeding after birth: an evaluation of simulation-based training in a low-resource setting. Acta Obstet Gynecol Scand. 2013 Dec 18;93(3):287–95.

31. Bartlett L, Cantor D, Lynam P, Kaur G, Rawlins B, Ricca J, et al. Facility-based active management of the third stage of labour: assessment of quality in six countries in sub-Saharan Africa. Bull World Health Organ. 2015 Nov 1;93(11):759–67.

32. Gohil JT, Tripathi B. A Study to Compare the Efficacy of Misoprostol, Oxytocin, Methyl-ergometrine and Ergometrine–Oxytocin in Reducing Blood Loss in Active Management of 3rd Stage of Labor. J Obstet Gynecol India. 2011 Aug 1;61(4):408.

33. Mukta M, Sahay PB. Role of Misoprostol 600 mcg Oral in Active Management of Third Stage of Labor: A Comparative Study with Oxytocin 10 IU i.m. J Obstet Gynecol India. 2013 Oct 1;63(5):325–7.

34. McCormick M.L., Sanghvi H.C.G., Kinzie B., McIntosh N. Preventing postpartum hemorrhage in low-resource settings. Int J Gynecol Obstet. 2002 Jun 7;77(3):267–75.

35. Nasr Ahmed, Shahin Ahmed Y., Elsamman Ali M., Zakherah Mahmoud S., Shaaban Omar M. Rectal misoprostol versus intravenous oxytocin for prevention of postpartum hemorrhage. Int J Gynecol Obstet. 2009 Feb 26;105(3):244–7.

36. Tan Jing, Cao Qiao, He Guo-lin, Cai Yu-han, Yu Jia-jie, Sun Xin, et al. Misoprostol versus ergometrine-oxytocin for preventing postpartum haemorrhage: a systematic review and meta-analysis of randomized controlled trials. J Evid-Based Med. 2016 Nov 27;9(4):194–204.

For peer review only

37. World Health Organization. WHO | WHO Recommendations on Prevention and Treatment of Postpartum Haemorrhage and the WOMAN Trial [Internet]. WHO. [cited 2018 Apr 2]. Available from: http://www.who.int/reproductivehealth/topics/maternal_perinatal/pph-woman-trial/en/

For peer review only



For peer review only - http://bmjopen.bmj.com/site/about/guidelines.xhtml

BMJ Open

# Access and use of oxytocin for post-partum hemorrhage prevention: a pre-post study targeting the poorest in six Mesoamerican countries

| | |
|---|---|
| Journal: | *BMJ Open* |
| Manuscript ID | bmjopen-2019-034084.R1 |
| Article Type: | Original research |
| Date Submitted by the Author: | 03-Dec-2019 |
| Complete List of Authors: | Kamath, Aruna; Institute for Health Metrics and Evaluation; University of Washington Department of Anesthesiology and Pain Medicine,<br>Schaefer, Alexandra; Institute for Health Metrics and Evaluation, Global Health<br>Palmisano, Erin; Institute for Health Metrics and Evaluation, Global Health<br>Johanns, Casey; Institute for Health Metrics and Evaluation, Global Health<br>Gonzalez Marmol, Alvaro; Inter-American Development Bank<br>Dinarte Mendoza, Mauricio ; Inter-American Development Bank<br>Schwarzbauer, Karla ; Inter-American Development Bank<br>Zúñiga-Brenes , Paola; Inter-American Development Bank<br>Ríos-Zertuche , Diego; Inter-American Development Bank<br>Iriarte, Emma; Inter-American Development Bank<br>mokdad, ali; Institute for Health Metrics and Evaluation<br>Hernandez Prado, Bernardo; Institute for Health Metrics and Evaluation |
| <b>Primary Subject Heading</b>: | Global health |
| Secondary Subject Heading: | Obstetrics and gynaecology, Public health |
| Keywords: | PUBLIC HEALTH, OBSTETRICS, International health services < HEALTH SERVICES ADMINISTRATION & MANAGEMENT |
| | |

SCHOLARONE™
Manuscripts

BMJ

*I, the Submitting Author has the right to grant and does grant on behalf of all authors of the Work (as defined in the below author licence), an exclusive licence and/or a non-exclusive licence for contributions from authors who are: i) UK Crown employees; ii) where BMJ has agreed a CC-BY licence shall apply, and/or iii) in accordance with the terms applicable for US Federal Government officers or employees acting as part of their official duties; on a worldwide, perpetual, irrevocable, royalty-free basis to BMJ Publishing Group Ltd ("BMJ") its licensees and where the relevant Journal is co-owned by BMJ to the co-owners of the Journal, to publish the Work in this journal and any other BMJ products and to exploit all rights, as set out in our licence.*

*The Submitting Author accepts and understands that any supply made under these terms is made by BMJ to the Submitting Author unless you are acting as an employee on behalf of your employer or a postgraduate student of an affiliated institution which is paying any applicable article publishing charge ("APC") for Open Access articles. Where the Submitting Author wishes to make the Work available on an Open Access basis (and intends to pay the relevant APC), the terms of reuse of such Open Access shall be governed by a Creative Commons licence – details of these licences and which Creative Commons licence will apply to this Work are set out in our licence referred to above.*

*Other than as permitted in any relevant BMJ Author's Self Archiving Policies, I confirm this Work has not been accepted for publication elsewhere, is not being considered for publication elsewhere and does not duplicate material already published. I confirm all authors consent to publication of this Work and authorise the granting of this licence.*

For peer review only

# Access and use of oxytocin for post-partum hemorrhage prevention: a pre-post study targeting the poorest in six Mesoamerican countries

Aruna M. Kamath,[1,2] Alexandra M. Schaefer,[1] Erin B. Palmisano,[1] Casey K. Johanns,[1] Alvaro Gonzalez Marmol,[3] Mauricio Dinarte Mendoza,[3] Karla Schwarzbauer,[3] Paola Zúñiga-Brenes,[3] Diego Rios-Zertuche,[3] Emma Iriarte,[3] Ali H. Mokdad,[1] Bernardo Hernández[1]

1. Institute for Health Metrics and Evaluation, University of Washington, Seattle, WA, USA
2. Department of Anesthesiology, University of Washington, Seattle, WA, USA
3. Salud Mesoamérica Initiative/Inter-American Development Bank, Panama, Panama; Tegucigalpa, Honduras; San José, Costa Rica; and Washington, DC, USA

**Corresponding Author:**

Aruna M. Kamath, MD, MPH
Institute for Health Metrics and Evaluation
University of Washington
2301 Fifth Ave, Suite 600
Seattle, WA 98121
USA
Tel: 206.897.2817
Email: akamath@uw.edu

For peer review only

# ABSTRACT

**Objectives:** Hemorrhage remains the leading cause of maternal mortality in Central America. The Salud Mesoamerica Initiative aims to reduce such mortality via performance indicators. Our objective was to assess the availability and administration of oxytocin, before and after applying Salud Mesoamerica Initiative interventions in the poorest health facilities across Central America.

**Design:** pre-post study.

**Setting:** 166 basic-level and comprehensive-level health facilities in Belize, Guatemala, Honduras, Mexico, Nicaragua, and Panama.

**Participants:** A random sample of medical records for uncomplicated full-term deliveries (n = 2,470) per international classification of diseases coding at baseline (July 2011–August 2013) and at first-phase follow-up (January 2014-October 2014).

**Interventions:** A year of intervention implementation prior to first-phase follow-up data collection focused on improving access to oxytocin by strengthening supply chains, procurement, storage practices, and pharmacy inventory monitoring, using a results-based financing model.

**Primary and secondary outcome measures:** Oxytocin availability (primary outcome) and administration (secondary outcome) for post-partum hemorrhage prevention.

**Results**: Availability of oxytocin increased from 82.9% to 97.6%. Oxytocin administration increased from 83.6% to 88.4%. Significant improvements were seen for availability of oxytocin (aOR = 8.41, 95% CI = 1.50–47.30). Administration of oxytocin was found to be significantly higher in Honduras (aOR = 2.96; 95% CI = 1.00-8.76) in reference to Guatemala at follow-up.

**Conclusion**: After interventions to increase health facility supplies, the study showed a significant improvement in availability but not administration of oxytocin in poor communities within Mesoamerica. Efforts are needed to improve the use of oxytocin.




For peer review only - http://bmjopen.bmj.com/site/about/guidelines.xhtml

For peer review only

**Trial registration:** not applicable

## STRENGTHS AND LIMITATIONS OF THIS STUDY

- Using a results-based financing model, interventions focused on improving access to oxytocin by strengthening supply chains, procurement, storage practices, and pharmacy inventory monitoring,
- Based on a random sampling method of medical records, a multinational dataset containing 2,470 deliveries was utilized for the pre-post evaluation of post-partum hemorrhage prevention in the poorest populations of Mesoamerica.
- To measure comparable metrics from varying medical record sources, indicators linked to ICD coding were constructed by the Ministries of Health based on protocols and guidelines of each country.
- We used multivariable logistic regression analysis to evaluate possible factors associated with the primary outcome of oxytocin availability and the secondary outcome of oxytocin administration.
- Challenges to this study involve logistic limitations in collecting data on some of the facility inputs, sample size variation by country, and quality of record keeping.

## INTRODUCTION

Hemorrhage remains the leading cause of maternal mortality both worldwide and in Mesoamerica, accounting for 31.4% of total maternal deaths globally(1) and ranging from 38.0% of total maternal deaths in Nicaragua and Guatemala to 17.9% in Belize based on 2016 Global Burden of Disease Study estimates.(2) While maternal mortality has declined in the last two decades, Millennium Development Goal 5, of reducing maternal mortality by three-fourths, was not achieved globally in 2015.(3,4) A new target has been set for the Sustainable Development Goals to reduce the global maternal mortality ratio to less than 70 per 100,000 live births by 2030.(5) Currently, most



For peer review only - http://bmjopen.bmj.com/site/about/guidelines.xhtml

of the Mesoamerican countries in this study fall short of or near this target, with the highest maternal mortality ratio, of 109.6 per 100,000 live births, occurring in Honduras.(6)

International guidelines for postpartum hemorrhage, from the World Health Organization (WHO), International Federation of Gynecology and Obstetrics (FIGO), and the International Confederation of Midwives (ICM), recommend active management of third stage of labor (AMTSL) with emphasis on administering oxytocin, the drug of choice for prevention and treatment of uterine atony.(7,8) Oxytocin is the cornerstone of AMTSL due to its efficacy in reducing postpartum hemorrhage risk by 40–60%.(9–11) Despite such evidence, translating these best practices into a real-world clinical standard of care is not optimally applied in Mesoamerica, with the proper use of uterotonics as low as 10–20% in some studies.(12,13)

With efforts to close the evidence-to-practice gap, the Salud Mesoamérica Initiative (SMI) aims to reduce maternal and child mortality for the poorest quintile in Mesoamerica.(14) SMI is a public-private partnership between the Ministries of Health in participating Mesoamerican countries and donors that focuses on improving four major domains of maternal and child health: preventive child health and vaccines; family planning; antenatal care and postpartum care; and Essential Obstetric and Newborn Care (EONC).(15) SMI follows a results-based financing model (16,17) in which Ministries of Health commit to achieve negotiated targets for eight to twelve performance indicators. Within the EONC domain, health facility performance indicators focused on the availability of equipment (i.e. resuscitation equipment, cesarean section kits, stethoscopes), essential medications (i.e. antibiotics, antihypertensives), and laboratory inputs (i.e. glucometer, cell counter). To receive the performance payment incentive, countries need to achieve at least eighty percent of targets. At the beginning of each phase, donors contribute approximately half the funding, with the rest from domestic sources.

The first phase of SMI interventions, presented in this study, focused on system readiness by increasing availability of inputs and improving norms. The second upcoming phase directs efforts

For peer review only



For peer review only - http://bmjopen.bmj.com/site/about/guidelines.xhtml

towards coverage and quality of care. Performance indicators for postpartum hemorrhage prevention involve increasing availability of oxytocin in this first phase and ensuring administration of oxytocin postpartum in the second phase.

In this analysis, we examine foremost the availability, and to a lesser extent the administration, of oxytocin for the prevention of postpartum hemorrhage at the first follow-up of the SMI intervention. As such, we assess the extent to which international guidelines are adhered to on the patient level for one aspect of essential obstetric care in poor Mesoamerica.

# METHODS

## Study setting and design

As part of the Salud Mesoamérica Initiative, 2,470 uncomplicated deliveries from 166 health facilities were included in this analysis to assess the availability and administration of oxytocin for the prevention of postpartum hemorrhage in the poorest Mesoamerican communities. These poorest communities were identified by the Initiative administrators based on census data, using as criteria to have the highest concentration of population in the lowest quintile of income. Data collection was conducted at the baseline (July 2011–August 2013) and the first-phase follow-up (January 2014–October2014) time periods.

The first phase, a year of intervention implementation prior to follow-up data collection, focused on supply-side performance indicators. For postpartum hemorrhage prevention, the performance indicator involved increasing the availability of oxytocin, through interventions such as strengthening supply chains (i.e. develop efficient distribution routes; optimize supply patterns and frequency of stocking facilities); improving procurement processes (i.e. review stock estimates for stockouts and emergency supplies; establish hospital policies to purchase life-saving medicines), warehouse and pharmacy storage practices (i.e. monitor continuously temperature and electricity



For peer review only - http://bmjopen.bmj.com/site/about/guidelines.xhtml

for cold chains); traffic light systems to monitor expiry dates (i.e. color code expiration dates on a monthly basis); and inventory management processes (i.e first-in first-out utilization of medicines; and anticipate emergency stock with each reorder cycle).

Facilities were classified into different levels of Essential Obstetric and Neonatal Care (EONC):(15) basic-level facilities manage uncomplicated vaginal deliveries and stabilize patients with complications prior to transfer to higher level of care; and comprehensive-level facilities oversee uncomplicated and complicated births, accept referral patients, and perform surgical and emergency care. While the project and its evaluation includes all countries in Mesoamerica, due to availability of data for this specific analysis we used data from Ministry of Health facilities serving the poorest areas in Belize, Guatemala, Honduras, Mexico (state of Chiapas), Nicaragua, and Panama. Basic- and comprehensive-level health facilities were selected with certainty due to a small number of these hospitals.

A three-part health facility survey was administered at these facilities for both rounds of data collection. The survey included an interview questionnaire to the facility directors on the facility infrastructure and resources; an observation checklist of pharmaceutical inventory and medical and laboratory equipment; and a review of medical records to examine care practices. Medical records of uncomplicated deliveries per international classification of diseases (ICD) coding during the specified timeframes were sampled at random. Following a systematic sampling method, records were extracted until the required quota for each facility level was met.

With the list of deliveries attended in each facility available, a random starting point was selected in time over the period of study (about 18 months). Cases were then selected with an interval equivalent to the sampling fraction. Sample size of records to be reviewed was calculated due to availability of resources, varying by round, facility, and country. Therefore, a larger overall sample size was assigned to countries with a larger operation (Honduras, Guatemala and Mexico), and was distributed across the facilities to be surveyed depending on their EONC level. Expected



For peer review only - http://bmjopen.bmj.com/site/about/guidelines.xhtml

sample sizes had enough power to detect differences in evaluation indicators, including treatment to deliveries according to the national norms. Due to availability of resources, it was possible to increase the number of cases in the sample in the follow-up to increase power, especially in the countries with a reduced number of cases in the baseline (Belize). Overall, we had a power over 80% to detect differences of 10 percentage points between baseline and follow-up for oxytocin availability in all countries excepting Belize and Nicaragua. Additional information of the methodology is available elsewhere.(14,17–19)

To measure standardized, replicable, and comparable metrics from varying medical record sources, indicators that were linked to discharge diagnosis and ICD coding were constructed by the Ministries of Health based on the protocols and guidelines of each country. Criteria checklists for each indicator differed by EONC facility level. These criteria checklists were then transformed into data points and conditional algorithms for data collection. During field visits, medical record data availability and measurability of criteria were assessed.(20) Data collection was conducted by trained physicians and nurses from the region, and data were submitted electronically using the survey software DatStat Illume.

Availability of oxytocin was defined as supply on the day of survey visit; and administration of oxytocin was defined as given intravenously or intramuscularly for postpartum hemorrhage prevention (excluding postpartum hemorrhage treatment). Uncomplicated delivery records selected for review included full-term (>37 weeks) gestational age at participating health facilities, including cesarean sections without complications per ICD coding. Deliveries with adverse outcomes per ICD coding that required hemorrhage treatment were excluded, due to collection under a separate survey module.

For peer review only

## Statistical analysis

We used multivariable logistic regression analysis to evaluate possible facility-level factors associated with the primary outcome of oxytocin availability and the secondary outcome of oxytocin administration. Covariates evaluated in this study were timing of data collection (baseline versus first-phase follow-up), country, and EONC facility type (basic versus comprehensive). Additionally for oxytocin administration, we examined relevant training within the last year (routine labor care, basic emergency obstetrical care, or maternal complications care); and oxytocin availability (day of survey visit), and skilled personnel (doctor, nurse). When fitting the regression models, skilled personnel was dropped from the model due to predicting success perfectly, meaning a skilled personnel was always present for the administration of oxytocin. The rate of missing data was 4.4% for oxytocin availability and 0.7% for oxytocin administration for the regression analysis. P values <0.05 were considered significant. We used Stata 14.2 (StataCorp LP, College Station, TX, USA) for the analysis.

## Patient and Public Involvement

Patients or the public were not involved in the design, conduct, reporting, or dissemination of our research.

# RESULTS

## Sample characteristics

Table 1 presents the medical records, patient, facility, and personnel characteristics. The majority of deliveries were in basic-level health facilities (64.3%). We sampled the most medical records from Guatemala (27.5%), followed by Chiapas (Mexico) (20.8%), Honduras (19.6%), Panama (16.0%), Nicaragua (11.9%), and Belize (4.2%), based on the quota for records within each facility, prevalence of records of interest, and sampling interval. With a range from 11 to 48 years, the mean



For peer review only - http://bmjopen.bmj.com/site/about/guidelines.xhtml

maternal age was 24.3 years. Of these patients, 55.2% were single (no social partnership), and 72.7% attained less than a secondary education. Nearly all patients (99.7%) had a single gestational pregnancy, and only 0.3% had a multiple gestational pregnancy. Most of these patients delivered vaginally (97.7%) as opposed to by cesarean section (2.3%). Supply (on the day of survey visit) for oxytocin at baseline was 82.9%, which increased to near complete availability (97.6%) at first-phase follow-up. Personnel on staff – that is, skilled birth attendants employed by the facility – at baseline was 96.2% for physicians and 91.0% for nurses and increased to 100% and 93.2%, respectively, at follow-up. Relevant training – that is, instruction on routine labor care, basic emergency obstetrical care, or maternal complications care – provided within the last year increased from 74.0% at baseline to 92.0% at first-phase follow-up.

## Table 1. Medical records, patient, and facility characteristics by first-phase follow-up

| **Medical records characteristics** | | | | |
|---|---|---|---|---|
| | | **n (%)** | | |
| | | **Baseline** 922 (37.3) | **Follow-up** 1,548 (62.7) | **Total** 2,470[a] (100) |
| EONC[b] facility type | | | | |
| | Basic-level | 616 (66.8) | 971 (62.7) | 1,587 (64.3) |
| | Comprehensive-level | 306 (33.2) | 577 (37.3) | 883 (35.8) |
| Country | | | | |
| | Belize | 14 (1.5) | 90 (5.8) | 104 (4.2) |
| | Guatemala | 247 (26.8) | 432 (27.9) | 679 (27.5) |
| | Honduras | 234 (25.4) | 249 (16.1) | 483 (19.6) |
| | Mexico | 180 (19.5) | 334 (21.6) | 514 (20.8) |
| | Nicaragua | 90 (9.8) | 205 (13.2) | 295 (11.9) |
| | Panama | 157 (17.0) | 238 (15.4) | 395 (16.0) |
| **Patient characteristics** | | | | |
| Maternal age[c] | | 24.3 (6.7) | 24.3 (6.6) | 24.3 (6.6) |
| Marital status | | | | |
| | Single | 409 (61.8) | 656 (51.8) | 1,065 (55.2) |
| | Partnership | 253 (38.2) | 610 (48.1) | 863 (44.8) |
| Education | | | | |
| | Less than secondary | 392 (70.5) | 772 (73.9) | 1,164 (72.7) |
| | Secondary or higher | 164 (29.5) | 273 (26.1) | 437 (27.3) |
| Pregnancy type | | | | |
| | Single gestational | 154 (98.7) | 917 (99.9) | 1,071 (99.7) |
| | Multiple gestational | 2 (1.3) | 1 (0.1) | 3 (0.3) |
| Delivery type | | | | |



For peer review only - http://bmjopen.bmj.com/site/about/guidelines.xhtml

| | | | | |
|---|---|---|---|---|
| | Vaginal | 409 (98.6) | 1,091 (97.3) | 1,500 (97.7) |
| | Cesarean section | 6 (1.5) | 30 (2.7) | 36 (2.3) |
| **Facility and personnel characteristics** | | | | |
| | | **n (%)** | | |
| | | **Baseline** 78 (47.0) | **Follow-up** 88 (53.0) | **Total** 166 (100) |
| | Oxytocin supply[d] | 58 (82.9) | 83 (97.6) | 141 (91.0) |
| | Personnel on staff[e] | | | |
| | Physician | 75 (96.2) | 88 (100) | 163 (98.2) |
| | Nurse | 71 (91.0) | 82 (93.2) | 153 (92.2) |
| | Relevant training[f] | 57 (74.0) | 81 (92.0) | 138 (83.6) |

a n may vary for each variable due to missingness

b Essential Obstetric and Newborn Care

c mean ± SD

d day of survey visit

e skilled birth attendants employed by the facility. Midwife excluded due to varying skill level amongst countries.

f within last year, includes routine labor care, basic emergency obstetrical care, maternal complications

## Availability and administration of oxytocin for postpartum hemorrhage prevention

Availability of oxytocin (on the day of the survey) increased from 82.9% to 97.7% and administration of oxytocin increased from 83.6% to 88.4% from baseline to first-phase follow-up. Notably, all countries achieved 100% availability of oxytocin at first-phase follow-up, except for Guatemala (91.7). Comprehensive level facilities achieved 100% availability, compared to basic level facilities (96.8%) at first-phase follow-up. Administration of oxytocin at first-phase follow-up ranged from 80.0% in Belize to 94.8% in Honduras. Oxytocin administration was nearly the same at basic (88.4%) and comprehensive level (88.5%) facilities at first-phase follow-up. (Table 2).

## Table 2. Oxytocin availability and administration by facility type, country, and first-phase follow-up

| **Oxytocin Availability** | | **n (%)** | | |
|---|---|---|---|---|
| | | **Baseline** 58 (82.9) | **Follow-up** 83 (97.6) | **Total[a]** 141 (91.0) |
| | EONC facility type[b] | | | |
| | Basic-level | 43 (84.3) | 61 (96.8) | 104 (91.2) |
| | Comprehensive-level | 15 (78.9) | 22 (100.0) | 37 (90.2) |
| | Country | | | |
| | Belize | 4 (100.0) | 4 (100.0) | 8 (100.0) |



For peer review only - http://bmjopen.bmj.com/site/about/guidelines.xhtml
For peer review only

| | | | |
|---|---|---|---|
| Guatemala | 15 (88.2) | 22 (91.7) | 37 (90.2) |
| Honduras | 13 (92.9) | 12 (100.0) | 25 (96.2) |
| Mexico | 7 (100.0) | 14 (100.0) | 21 (75.0) |
| Nicaragua | 6 (85.7) | 14 (100.0) | 20 (95.2) |
| Panama | 13 (92.9) | 17 (100.0) | 30 (96.8) |
| **Oxytocin Administration for prevention of post-partum hemorrhage** | **n (%)** | | |
| | **Baseline** 771 (83.6) | **Follow-up** 1,353 (88.4) | **Total** 2,124 (86.6) |
| EONC facility type | | | |
| Basic-level | 521 (84.6) | 852 (88.4) | 1,373 (86.9) |
| Comprehensive-level | 250 (81.7) | 501 (88.5) | 751 (86.1) |
| Country | | | |
| Belize | 9 (64.3) | 72 (80.0) | 81 (77.9) |
| Guatemala | 198 (80.2) | 393 (91.0) | 591 (87.0) |
| Honduras | 224 (95.7) | 221 (94.8) | 445 (95.3) |
| Mexico | 129 (71.7) | 277 (82.9) | 406 (79.0) |
| Nicaragua | 87 (96.7) | 168 (82.8) | 255 (87.0) |
| Panama | 124 (79.0) | 222 (93.3) | 346 (87.6) |

a n may vary for each variable due to missingness

b Essential Obstetric and Newborn Care

For peer review only

Table 3 shows possible first-phase follow-up and facility determinants of the availability (primary outcome) and administration of oxytocin (secondary outcome). Odds of availability were significantly higher at first-phase follow-up compared to baseline (aOR = 8.41, 95% CI = 1.50–47.30), while odds of administration increased in magnitude but were not significant at first-phase follow-up (aOR = 1.63, 95% CI 0.83–3.21). Additionally, administration of oxytocin was found to be significantly higher in Honduras (aOR = 2.96; 95% CI = 1.00–8.76) in reference to Guatemala. Other covariates examined, such as comprehensive-level facility, relevant training, and oxytocin availability, showed a positive but not significant correlation to the outcomes.

## Table 3. Factors associated with availability (primary outcome) and administration of oxytocin (secondary outcome)

| | **Oxytocin availability** | | **Oxytocin administration for prevention of post-partum hemorrhage** | |
|---|---|---|---|---|
| | **Crude OR** (95% CI) (n= 2,257) | **Adjusted OR[a]** (95% CI) (n= 2,257) | **Crude OR** (95% CI) (n =2,343) | **Adjusted OR** (95% CI) (n =2,343) |
| First-phase follow-up | **7.29 (1.48–35.84)** | **8.41 (1.50–47.30)** | 1.43 (0.79–2.62) | 1.63 (0.83–3.21) |
| Country | | | | |



For peer review only - http://bmjopen.bmj.com/site/about/guidelines.xhtml

| | | | | |
|---|---|---|---|---|
| Belize | *Predicts success perfectly[b]* | *Predicts success perfectly* | 0.52 (0.13–2.12) | 0.44 (0.11–1.71) |
| Guatemala | 1 | 1 | 1 | 1 |
| Honduras | 1.70 (0.18–16.46) | 2.24 (0.27–18.24) | **2.71 (1.02–7.22)** | **2.96 (1.00–8.76)** |
| Mexico | 0.67 (0.14–3.10) | 0.51 (0.11–2.46) | 0.57 (0.25–1.31) | 0.57 (0.24–1.37) |
| Nicaragua | 2.22 (0.23–21.71) | 2.01 (0.19–20.92) | 1.00 (0.38–2.62) | 0.95 (0.35–2.54) |
| Panama | 3.43 (0.36–33.05) | 3.83 (0.36–41.22) | 1.04 (0.41–2.66) | 1.03 (0.40–2.61) |
| Comprehensive-level facility | 1.00 (0.24–4.15) | 1.29 (0.31–5.31) | 0.92 (0.47–1.81) | 0.96 (0.46–2.00) |
| Relevant training[c] | -- | -- | 1.49 (0.71–3.13) | 1.08 (0.44–2.67) |
| Oxytocin availability[d] | -- | -- | 2.01 (0.92–4.39) | 1.45 (0.66–3.19) |

a adjusted for first-phase follow-up, country, and comprehensive-level facility. Relevant training and oxytocin availability not included (not applicable).

b Belize attained availability of oxytocin at all times (regression for this binary variable not possible)

c within last year, includes routine labor care, basic emergency obstetrical care, maternal complications

d day of survey visit

All models clustered at health facility level

# DISCUSSION

There remains a paucity of studies with regard to oxytocin access and use in Mesoamerica (13,21–23) or in low-resource countries in general.(12,24–30) In Mesoamerica, García-Elorrio et al 2014 and Low et al 2012 conducted pre-post evaluations at health facilities within a single country, rather than a multinational analysis. These studies focused on oxytocin administration interventions, such as multifaceted provider skills in Nicaragua and AMTSL training in Honduras respectively, without attention to improving oxytocin availability, the primary outcome of this study. Therefore, we provide an updated, regional evaluation of postpartum hemorrhage prevention practices that may guide clinical management and policy priorities for this targeted population. Our study showed that the availability of oxytocin improved significantly after system readiness interventions, among the poorest population within Mesoamerica.

This analysis attests that clinical standards of care could be achieved in Mesoamerica with proper monitoring and management of oxytocin supplies. As expected from the implementation

For peer review only

timeline, the first stage of the Salud Mesoamérica Initiative focused on improving system readiness, namely strengthening facility infrastructure, supply chains, procurement processes, storage practices, and inventory monitoring of drugs and equipment.(14,16) Consequently, increasing the availability of oxytocin, the primary goal of the first phase of the initiative, was achieved. While, administration of oxytocin, a lesser goal, increased but not in the same proportion as availability, which suggests that increasing the availability of inputs is necessary but not sufficient to improve quality of care. Comprehensive quality improvement strategies would be needed that involve not only increasing availability of equipment and supplies, but also improving skills and abilities of personnel, and establishing mechanisms to improve processes, identify causes, and lift barriers to ensure proper practices of care in the next phase.

This study has some limitations. First, oxytocin availability was defined based on the day of the visit to health facilities, which may not match the day of delivery in a medical record. Due to logistic limitations, it was not possible to assess stock-outs of oxytocin over a longer period of time. Therefore, we must consider oxytocin availability the day of the visit as a proxy for availability over a longer period of time. Quality of available oxytocin cannot be included when defining oxytocin availability, as data was not collected on expiration or storage of oxytocin. Second, this study focused on uncomplicated full-term deliveries and did not include patients resulting in hemorrhage due to two separate survey modules for data collection. In the module with adverse outcomes from hemorrhage, uterotonics were often administered more than once without distinction of whether for prevention or treatment. Third, sample sizes also vary by country, and in some cases like Belize, the reduced sample size limited our capacity to have country-specific precise estimates and to detect significant differences between baseline and follow-up measurements. Fourth, quality of record keeping may affect the information used in this study and may vary by country. Nonetheless, we used a standard methodology across countries, automated data collection, and used quality control measures that ensure comparability between countries.

For peer review only

## CONCLUSION

The study showed a significant improvement in availability of oxytocin at facilities that provide care to poor communities within Mesoamerica, but not optimal administration, as expected after emphasis on supply-side interventions. Continued monitoring and evaluation, beyond input availability, are essential to better understand how to improve processes and clinical practices. Our study provides valuable information to close the evidence-to-practice gap and to reduce maternal mortality in Mesoamerica.

## LIST OF ABBREVIATIONS

Active management of third stage of labor (AMTSL)

Essential Obstetric and Newborn Care (EONC)

International Confederation of Midwives (ICM)

International Federation of Gynecology and Obstetrics (FIGO)

Salud Mesoamérica Initiative (SMI)

World Health Organization (WHO)

## DECLARATIONS

### Ethics approval

Approval for this study involved the institutional review board from the University of Washington (exemption as a non-human-subject research determination), partnering data-collection agencies (El Colegio de la Frontera Sur-Mexico), the Ministry of Health in each country (Belize, Guatemala, Honduras, Mexico, Nicaragua, Panama), and the indigenous communities in Panama and Mexico. Prior to data collection, informed consent was obtained from each health facility

For peer review only



For peer review only - http://bmjopen.bmj.com/site/about/guidelines.xhtml

administrator. During the data extraction process, medical records were anonymized. No patient consent required.

## Data Sharing

Data are owned by the Inter-American Development Bank. SMI baseline survey data, which comprise the minimal dataset, are publicly available on the SMI website. SMI follow-up survey data are currently available upon written request from the Inter-American Development Bank, whose contact information is listed below. Javier Lesaca, Communications Officer, Salud Mesoamerica Initiative, Inter-American Development Bank, 1300 New York Ave. NW, Washington, DC 20577. contact@saludmesoamerica.org.

## Competing interests

The authors declare that they have no competing interests

## Funding

Salud Mesoamérica Initiative (SMI) is a public-private partnership funded by the Bill & Melinda Gates Foundation (Grant Number OPPGH5328), Agencia Española de Cooperación Internacional para el Desarrollo (10.13039/501100004892), and the Carlos Slim Foundation (10.13039/100004429), through the Inter-American Development Bank. AMK is funded by the National Institutes of Health T32 grant (T32GM086270). Funders had no role in study design, data collection, data analysis and interpretation, or preparation of the manuscript.

## Authors' contributions

AMK conceived the study and prepared data analysis, interpretation of data, initial draft, and final manuscript. AMS, EBP, and CKJ contributed to and reviewed data analysis. AGM, MDM, KS, PZB, DRZ, and EI contributed to the acquisition of the data. BH and AHM supervised the study and data compilation and contributed to interpretation of data. All authors critically reviewed the draft and approved the final manuscript.

For peer review only



For peer review only - http://bmjopen.bmj.com/site/about/guidelines.xhtml

## Acknowledgments

The authors recognize our colleagues at the Inter-American Development Bank, the Institute for Health Metrics and Evaluation, and our collaborating data collection institutions across Mesoamerica: University of Belize in Belize, Fundación FES in Guatemala and Honduras, El Colegio de la Frontera Sur in Mexico and Nicaragua, Centro de Investigación y Estudios en Salud/Universidad de Nicaragua in Nicaragua, and UNIMER-Centroamerica in Guatemala, Mexico, Honduras, Nicaragua, Panama, and Belize. We also acknowledge Adrienne Chew from the Institute for Health Metrics and Evaluations for editing this manuscript.

## REFERENCES

1. GBD 2016 Causes of Death Collaborators. Global, regional, and national age-sex specific mortality for 264 causes of death, 1980–2016: a systematic analysis for the Global Burden of Disease Study 2016. The Lancet. 2017 Sep 16;390(10100):1151–210.

2. Institute for Health Metrics and Evaluation (IHME). GBDCompareDataVisualization. Seattle, WA: IHME, University of Washington, 2016. [Internet]. [cited 2018 Aug 22]. Available from: http://vizhub.healthdata.org/gbd-compare.

3. United Nations. Millennium Development Goals Report 2013. [Internet]. 2013 [cited 2018 Mar 11]. Available from: http://www.un.org/millenniumgoals/pdf/report-2013/mdg-report-2013-english.pdf

4. Lozano R, Wang H, Foreman KJ, Rajaratnam JK, Naghavi M, Marcus JR, et al. Progress towards Millennium Development Goals 4 and 5 on maternal and child mortality: an updated systematic analysis. The Lancet. 2011 Sep 24;378(9797):1139–65.

5. Every Women Every Child. The global strategy for women's, children's, and adolescents' health (2016-2030) [Internet]. 2015 [cited 2018 Mar 11]. Available from: http://globalstrategy.everywomaneverychild.org/pdf/EWEC_globalstrategyreport_200915_FINAL_WEB.pdf

6. Kassebaum NJ, Barber RM, Bhutta ZA, Dandona L, Gething PW, Hay SI, et al. Global, regional, and national levels of maternal mortality, 1990–2015: a systematic analysis for the Global Burden of Disease Study 2015. The Lancet. 2016 Oct 8;388(10053):1775–812.

7. World Health Organization. WHO recommendations for the prevention and treatment of postpartum haemorrhage. [Internet]. 2012 [cited 2018 Mar 11]. Available from: http://apps.who.int/iris/bitstream/10665/75411/1/9789241548502_eng.pdf

8. Lalonde A, FIGO Safe Motherhood and Newborn Health (SMNH) Committee. Prevention and treatment of postpartum hemorrhage in low-resource settings. Int J Gynecol Obstet. 2012 May 1;117(2):108–18.



For peer review only - http://bmjopen.bmj.com/site/about/guidelines.xhtml

9. Prata N, Bell S, Weidert K. Prevention of postpartum hemorrhage in low-resource settings: current perspectives. Int J Womens Health. 2013;5:737–52.

10. Prendiville WJ, Harding JE, Elbourne DR, Stirrat GM. The Bristol third stage trial: active versus physiological management of third stage of labour. BMJ. 1988 Nov 19;297(6659):1295–300.

11. Elbourne DR, Prendiville WJ, Carroli G, Wood J, McDonald S. Prophylactic use of oxytocin in the third stage of labour. Cochrane Database Syst Rev. 2001;(4):CD001808.

12. Althabe F, Mazzoni A, Cafferata ML, Gibbons L, Karolinski A, Armbruster D, et al. Using Uniject to increase the use of prophylactic oxytocin for management of the third stage of labor in Latin America. Int J Gynaecol Obstet Off Organ Int Fed Gynaecol Obstet. 2011 Aug;114(2):184–9.

13. García-Elorrio E, Aleman A, Cafferata ML, Colomar M, Tomasso G, Lacayo Y, et al. A multifaceted intervention to increase prophylactic oxytocin use during the third stage of labor and to reduce routine episiotomies in Nicaragua. Int J Gynaecol Obstet Off Organ Int Fed Gynaecol Obstet. 2014 Oct;127(1):31–4.

14. Mokdad AH, Colson KE, Zúñiga-Brenes P, Ríos-Zertuche D, Palmisano EB, Alfaro-Porras E, et al. Salud Mesoamérica 2015 Initiative: design, implementation, and baseline findings. Popul Health Metr. 2015 Feb 7;13:3.

15. USAID MCHIP Essential Obstetric and Newborn Care (EONC) Program Implementation Guidance [Internet]. [cited 2018 Mar 20]. Available from: https://www.k4health.org/sites/default/files/EONC%20Program%20Implementation%20Guide%20v1.1.pdf

16. Regalia F, Eichler R, Gigli S, Cleason M, Tapia Conyer R, Hernandez G, et al. Not Your Cookie-Cutter Results-Based Aid Initiative: Salud Mesoamerica Initiative's Experience Improving Health for the Poorest in Mesoamerica [Internet]. Inter-American Development Bank; 2017 Oct [cited 2018 Jun 5]. Available from: http://publications.iadb.org/handle/11319/8567

17. Álvarez MC, Tapia Conyer R, Regalia F, Eichler R, Ciria Matilla MC, Nelson J, et al. External Measurement as a Catalyst for Change in a Regional Results-Based Aid Initiative - The Salud Mesoamerica Experience [Internet]. Inter-American Development Bank; 2017 Oct [cited 2018 Dec 11]. Available from: https://publications.iadb.org/handle/11319/8565

18. Mokdad AH, Palmisano EB, Zúñiga-Brenes P, Ríos-Zertuche D, Johanns CK, Schaefer A, et al. Supply-side interventions to improve health: Findings from the Salud Mesoamérica Initiative. PloS One. 2018;13(4):e0195292.

19. Duber HC, Hartford EA, Schaefer AM, Johanns CK, Colombara DV, Iriarte E, et al. Appropriate and timely antibiotic administration for neonatal sepsis in Mesoamérica. BMJ Glob Health. 2018 May 1;3(3):e000650.

20. Rios-Zertuche D, Zúñiga-Brenes P, Palmisano E, Hernández B, Schaefer A, Johanns CK, et al. Methods to measure quality of care and quality indicators through health facility surveys in low- and middle-income countries. Int J Qual Health Care [Internet]. [cited 2018 Dec 11]; Available from: https://academic-oup-com.offcampus.lib.washington.edu/intqhc/advance-article/doi/10.1093/intqhc/mzy136/5039910

For peer review only

21. Low Lisa Kane, Bailey Joanne Motino, Sacks Emma, Robles Chayla, Medina Lilian. Reduced postpartum hemorrhage after implementation of active management of the third stage of labor in rural Honduras. Int J Gynecol Obstet. 2012 Sep 12;119(3):217–20.

22. Dresang Lee T., González María Mercedes Ancheta, Beasley John, Bustillo Maura Carolina, Damos Jim, Deutchman Mark, et al. The impact of Advanced Life Support in Obstetrics (ALSO) training in low-resource countries. Int J Gynecol Obstet. 2015 Aug 4;131(2):209–15.

23. Walton Anna, Kestler Edgar, Dettinger Julia C., Zelek Sarah, Holme Francesca, Walker Dilys. Impact of a low-technology simulation-based obstetric and newborn care training scheme on non-emergency delivery practices in Guatemala. Int J Gynecol Obstet. 2015 Dec 11;132(3):359–64.

24. Figueras Albert, Narváez Edgar, Valsecia Mabel, Vásquez Susana, Rojas Germán, Camilo Angiolina, et al. An Education and Motivation Intervention to Change Clinical Management of the Third Stage of Labor—The GIRMMAHP Initiative. Birth. 2008 Nov 18;35(4):283–90.

25. Sheldon W, Blum J, Vogel J, Souza J, Gülmezoglu A, Winikoff B, et al. Postpartum haemorrhage management, risks, and maternal outcomes: findings from the World Health Organization Multicountry Survey on Maternal and Newborn Health. BJOG Int J Obstet Gynaecol. 2014 Mar 1;121:5–13.

26. Olmedo Benjamin, Miranda Eva, Cordon Oscar, Pettker Christian M., Funai Edmund F. Improving maternal health and safety through adherence to postpartum hemorrhage protocol in Latin America. Int J Gynecol Obstet. 2014 Jan 24;125(2):162–5.

27. Braddick Louise, Tuckey Victoria, Abbas Zan, Lissauer David, Ismail Khaled, Manaseki-Holland Semira, et al. A mixed-methods study of barriers and facilitators to the implementation of postpartum hemorrhage guidelines in Uganda. Int J Gynecol Obstet. 2015 Sep 28;132(1):89–93.

28. Hoogenboom G, Thwin MM, Velink K, Baaijens M, Charrunwatthana P, Nosten F, et al. Quality of intrapartum care by skilled birth attendants in a refugee clinic on the Thai-Myanmar border: a survey using WHO Safe Motherhood Needs Assessment. BMC Pregnancy Childbirth. 2015 Feb 5;15:17.

29. Nelissen Ellen, Ersdal Hege, Østergaard Doris, Mduma Estomih, Broerse Jacqueline, Evjen-Olsen Bjørg, et al. Helping mothers survive bleeding after birth: an evaluation of simulation-based training in a low-resource setting. Acta Obstet Gynecol Scand. 2013 Dec 18;93(3):287–95.

30. Bartlett L, Cantor D, Lynam P, Kaur G, Rawlins B, Ricca J, et al. Facility-based active management of the third stage of labour: assessment of quality in six countries in sub-Saharan Africa. Bull World Health Organ. 2015 Nov 1;93(11):759–67.



For peer review only - http://bmjopen.bmj.com/site/about/guidelines.xhtml

STROBE Statement—checklist of items that should be included in reports of observational studies

| | Item No | Recommendation |
|---|---|---|
| **Title and abstract** | 1 | (*a*) Indicate the study's design with a commonly used term in the title or the abstract p.1/line 1; p.2/line 32 |
| | | (*b*) Provide in the abstract an informative and balanced summary of what was done and what was found p.2/line 38-40; p.3/line 53-56. |
| **Introduction** | | |
| Background/rationale | 2 | Explain the scientific background and rationale for the investigation being reported p.3-5/lines 69-103. |
| Objectives | 3 | State specific objectives, including any prespecified hypotheses p.5/lines104-106. |
| **Methods** | | |
| Study design | 4 | Present key elements of study design early in the paper p.5-6/lines 118-127. |
| Setting | 5 | Describe the setting, locations, and relevant dates, including periods of recruitment, exposure, follow-up, and data collection p.5/lines 111-117. p.6/lines 129-138. |
| Participants | 6 | (*a*) *Cohort study*—Give the eligibility criteria, and the sources and methods of selection of participants. Describe methods of follow-up<br>*Case-control study*—Give the eligibility criteria, and the sources and methods of case ascertainment and control selection. Give the rationale for the choice of cases and controls<br>*Cross-sectional study*—Give the eligibility criteria, and the sources and methods of selection of participants p.6-7/lines 139-153. |
| | | (*b*) *Cohort study*—For matched studies, give matching criteria and number of exposed and unexposed<br>*Case-control study*—For matched studies, give matching criteria and the number of controls per case |
| Variables | 7 | Clearly define all outcomes, exposures, predictors, potential confounders, and effect modifiers. Give diagnostic criteria, if applicable p.7/lines169-173 |
| Data sources/ measurement | 8* | For each variable of interest, give sources of data and details of methods of assessment (measurement). Describe comparability of assessment methods if there is more than one group p.7/lines 160-167 |
| Bias | 9 | Describe any efforts to address potential sources of bias p. 14/lines 293-302 |
| Study size | 10 | Explain how the study size was arrived at p.7/lines 153-159. |
| Quantitative variables | 11 | Explain how quantitative variables were handled in the analyses. If applicable, describe which groupings were chosen and why p.6/lines 129-138; p.7-8/lines 169-175. |
| Statistical methods | 12 | (*a*) Describe all statistical methods, including those used to control for confounding p.8/lines 182-193. |
| | | (*b*) Describe any methods used to examine subgroups and interactions |
| | | (*c*) Explain how missing data were addressed p.8line 191 |
| | | (*d*) *Cohort study*—If applicable, explain how loss to follow-up was addressed<br>*Case-control study*—If applicable, explain how matching of cases and controls was addressed<br>*Cross-sectional study*—If applicable, describe analytical methods taking account of sampling strategy p.5/lines111-117, p.6-7/lines 146-159. |
| | | (*e*) Describe any sensitivity analyses not applicable |



For peer review only - http://bmjopen.bmj.com/site/about/guidelines.xhtml

| | | |
|---|---|---|
| **Results** | | |
| Participants | 13* | (a) Report numbers of individuals at each stage of study—eg numbers potentially eligible, examined for eligibility, confirmed eligible, included in the study, completing follow-up, and analysed p.9/line 213 (Table 1) |
| | | (b) Give reasons for non-participation at each stage not applicable |
| | | (c) Consider use of a flow diagram |
| Descriptive data | 14* | (a) Give characteristics of study participants (eg demographic, clinical, social) and information on exposures and potential confounders p.9/line 213 (Table 1) |
| | | (b) Indicate number of participants with missing data for each variable of interest p.9/line 213 (Table 1); p.8/lines 190-192. |
| | | (c) *Cohort study*—Summarise follow-up time (eg, average and total amount) not applicable |
| Outcome data | 15* | *Cohort study*—Report numbers of outcome events or summary measures over time |
| | | *Case-control study*—Report numbers in each exposure category, or summary measures of exposure |
| | | *Cross-sectional study*—Report numbers of outcome events or summary measures p.10/line 230 (Table 2) |
| Main results | 16 | (*a*) Give unadjusted estimates and, if applicable, confounder-adjusted estimates and their precision (eg, 95% confidence interval). Make clear which confounders were adjusted for and why they were included p.11/line 243 (Table 3) |
| | | (*b*) Report category boundaries when continuous variables were categorized not applicable |
| | | (*c*) If relevant, consider translating estimates of relative risk into absolute risk for a meaningful time period not applicable |
| Other analyses | 17 | Report other analyses done—eg analyses of subgroups and interactions, and sensitivity analyses not applicable |
| **Discussion** | | |
| Key results | 18 | Summarise key results with reference to study objectives p. 12/lines 260-262. |
| Limitations | 19 | Discuss limitations of the study, taking into account sources of potential bias or imprecision. Discuss both direction and magnitude of any potential bias p.14/lines 293-307. |
| Interpretation | 20 | Give a cautious overall interpretation of results considering objectives, limitations, multiplicity of analyses, results from similar studies, and other relevant evidence p.13-14/lines 276-291 |
| Generalisability | 21 | Discuss the generalisability (external validity) of the study results p.14/lines 309-311. |
| **Other information** | | |
| Funding | 22 | Give the source of funding and the role of the funders for the present study and, if applicable, for the original study on which the present article is based p.16/lines 344-349 |

*Give information separately for cases and controls in case-control studies and, if applicable, for exposed and unexposed groups in cohort and cross-sectional studies.

**Note:** An Explanation and Elaboration article discusses each checklist item and gives methodological background and published examples of transparent reporting. The STROBE checklist is best used in conjunction with this article (freely available on the Web sites of PLoS Medicine at http://www.plosmedicine.org/, Annals of Internal Medicine at http://www.annals.org/, and Epidemiology at http://www.epidem.com/). Information on the STROBE Initiative is available at www.strobe-statement.org.


For peer review only - http://bmjopen.bmj.com/site/about/guidelines.xhtml
